# Selenoprotein N is dynamically expressed during mouse development and detected early in muscle precursors

**DOI:** 10.1186/1471-213X-9-46

**Published:** 2009-08-22

**Authors:** Perrine Castets, Svetlana Maugenre, Corine Gartioux, Mathieu Rederstorff, Alain Krol, Alain Lescure, Shahragim Tajbakhsh, Valérie Allamand, Pascale Guicheney

**Affiliations:** 1 Inserm, U582, F-75013 Paris, France; 2 UPMC Univ Paris 06, UMR_S582, Institut de Myologie, IFR14, F-75013 Paris, France; 3 Architecture et Réactivité de l'ARN, Université de Strasbourg, CNRS, IBMC, F-67084 Strasbourg, France; 4 CNRS, URA 2578, Institut Pasteur, F-75015 Paris, France; 5 AP-HP, Groupe Hospitalier Pitié-Salpêtrière, Service de Biochimie Métabolique, F-75013 Paris, France; 6 Inserm, U956, UPMC Univ Paris 06, UMR_S956, F-75013 Paris, France; 7 Inserm, U974, Institut de Myologie, CNRS UMR7215, UPMC Univ Paris 06, UMR_S974, IFR14, F-75013 Paris, France; 8 Innsbruck Medical University, Biocenter, Section for Genomics and RNomics, A-6020 Innsbruck, Austria

## Abstract

**Background:**

In humans, mutations in the *SEPN1 *gene, encoding selenoprotein N (SelN), are involved in early onset recessive neuromuscular disorders, referred to as *SEPN1*-related-myopathies. The mechanisms behind these pathologies are poorly understood since the function of SelN remains elusive. However, previous results obtained in humans and more recently in zebrafish pointed to a potential role for SelN during embryogenesis. Using qRT-PCR, Western blot and whole mount *in situ *hybridization, we characterized in detail the spatio-temporal expression pattern of the murine *Sepn1 *gene during development, focusing particularly on skeletal muscles.

**Results:**

In whole embryos, *Sepn1 *transcripts were detected as early as E5.5, with expression levels peaking at E12.5, and then strongly decreasing until birth. In isolated tissues, only mild transcriptional variations were observed during development, whereas a striking reduction of the protein expression was detected during the perinatal period. Furthermore, we demonstrated that *Sepn1 *is expressed early in somites and restricted to the myotome, the sub-ectodermal mesenchyme and the dorsal root ganglia at mid-gestation stages. Interestingly, *Sepn1 *deficiency did not alter somitogenesis in embryos, suggesting that SelN is dispensable for these processes in mouse.

**Conclusion:**

We characterized for the first time the expression pattern of *Sepn1 *during mammalian embryogenesis and we demonstrated that its differential expression is most likely dependent on major post-transcriptional regulations. Overall, our data strongly suggest a potential role for selenoprotein N from mid-gestation stages to the perinatal period. Interestingly, its specific expression pattern could be related to the current hypothesis that selenoprotein N may regulate the activity of the ryanodine receptors.

## Background

Selenium is a rare trace element mainly present in biological systems as a selenocysteine (Sec), an amino acid found in proteins called selenoproteins and encountered in all lineages of life. Insertion of the Sec residue occurs through a recoded UGA stop codon which is recognized by a complex machinery involving a secondary structure present in the 3'UTR region of the mRNA (Sec Insertion Sequence, SECIS), which interacts during translation with specific factors, such as SBP2 (SECIS Binding Protein 2) and eEFSec (eukaryote Elongation Factor), leading to Sec insertion rather than termination [1,2]. In mammals, about 25 selenoproteins have been described [3]. Most of them are enzymes involved in oxidation-reduction reactions, with the selenocysteine residue(s) usually located in the catalytic site and conferring a strong enzymatic reactivity [4,5]. Moreover, most selenoproteins are expressed early during development [2,6] and for several of them an essential role in embryogenesis has been established [7-9]. Selenium deficiency has been associated with different syndromes, such as Keshan cardiomyopathy, white muscle disease or rigid lamb syndrome, leading to dietary intake recommendations for humans and livestock [10]. Notably, mutations were identified in the *SEPN1 *gene, encoding selenoprotein N (SelN), as the genetic cause for Rigid Spine Muscular Dystrophy (*RSMD1*) [11]. This pathology is characterized by axial weakness, severe scoliosis usually requiring surgery, and respiratory insufficiency due to respiratory muscles weakness and necessitating mechanical nocturnal ventilation [12]. *SEPN1 *mutations were subsequently associated with three other neuromuscular disorders: the classical form of Multi-minicore Disease (*MmD*) [13], rare cases of Desmin-Related Myopathy with Mallory Body-like Inclusions (*MB-DRM*) [14] and of Congenital Fibre Type Disproportion (*CFTD*) [15]. These four early onset autosomal recessive pathologies exhibit clinical and morphological overlaps; they are now grouped and termed *SEPN1*-related myopathies (*SEPN1*-RM).

We showed previously that SelN is a glycoprotein located in the membrane of the endoplasmic reticulum [16]. In humans, its expression appeared ubiquitous and was down-regulated during myoblast differentiation in culture, and in the transition from foetal to adult tissues [16]. In zebrafish, an early expression of *sepn1 *was shown during embryogenesis, specifically in somites, the tail bud and the notochord [6,17]. Furthermore, *sepn1 *zebrafish mutants obtained by morpholino injection, exhibited strong developmental defects such as tail malformations, disorganisation of somite borders, and abnormalities in muscles development and architecture [17,18]. These data, as well as the early onset of the human pathology, clearly pointed to a possible role for SelN during muscle development. Interestingly, physical and functional interactions between SelN and ryanodine receptors (RyR) were recently demonstrated, indicating that SelN may regulate RyR activity in muscles [17,19].

Here, we characterized extensively the expression pattern of *Sepn1 *in mouse during pre- and post-natal development, in several tissues, particularly in skeletal muscles. We demonstrated that *Sepn1 *is expressed early during mouse embryogenesis and that it is restricted to specific areas, including muscle precursors, at mid-gestation stages. In isolated tissues, mild variations of *Sepn1 *transcripts were detected between ages, whereas the protein expression was strikingly down-regulated during the perinatal period, indicating post-transcriptional regulations of *Sepn1 *expression during development.

## Results

### Spatio-temporal dynamics of *Sepn1 *expression

**Figure 1:**
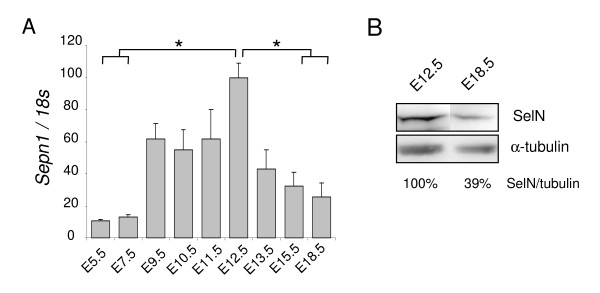
***Sepn1 *expression in whole embryos**. **A: ***Sepn1 *expression was quantified by qRT-PCR on cDNA from whole embryos between E5.5 and E18.5. Normalization is performed on the *18s *gene. Expression was detected as early as E5.5 and strongly increased until E12.5. From this stage until birth, a striking decrease of the expression was observed. *, p < 0.05. B: Western blot analysis on 60 μg of proteins from E12.5 and E18.5 embryos was carried out using SelN and α-tubulin (normalization) antibodies. SelN expression was reduced more than two fold at E18.5 compared to E12.5.

In whole embryos, *Sepn1 *expression was detected by qRT-PCR as early as E5.5, with higher levels from E9.5 to E12.5 (six and ten fold increase, respectively). We then observed a five fold reduction of the expression from E12.5 to E18.5 (Figure 1A). This decrease was confirmed at the protein level by Western blot since SelN was reduced by more than two fold in E18.5 embryos compared to E12.5 (Figure 1B).

**Figure 2:**
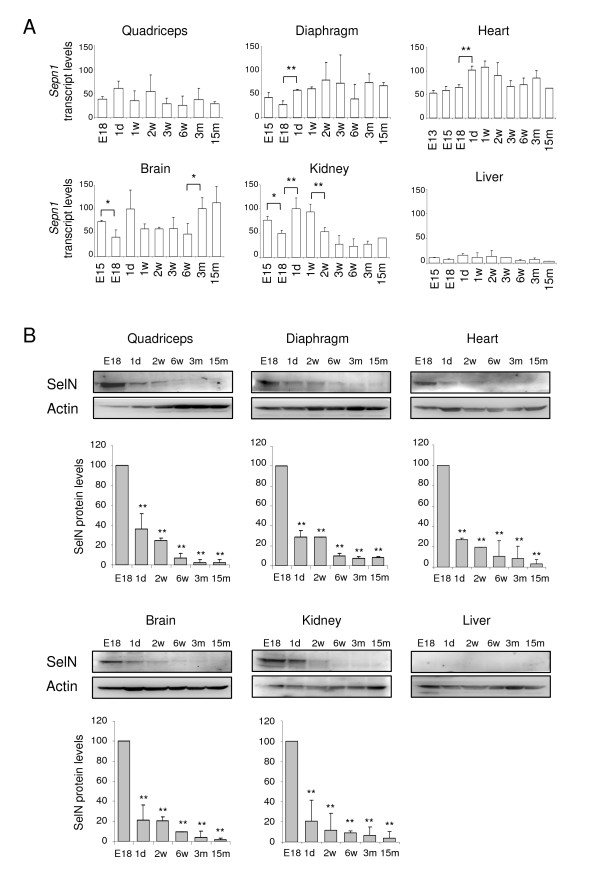
**Expression of *Sepn1 *transcript and SelN protein in isolated tissues between late embryonic and post-natal stages**. **A: ***Sepn1 *expression was quantified by qRT-PCR and normalized to the *18s *gene. Expression in quadriceps was quantified from E18 to 15 months; in diaphragm, liver, brain and kidney from E15 to 15 months; in heart from E13 to 15 months. Only mild variations were detected, including between pre- and postnatal stages. Significant variations are indicated for two consecutive points: *, p < 0.05; **, p < 0.01. B: SelN Western blot analyses are shown for quadriceps, diaphragm, heart, liver, brain and kidney at stages E18, postnatal day 1, 2 and 6 weeks, 3 and 15 months. SelN was undetectable in liver. Actin was used as a reference. Quantifications were normalized on the expression measured at E18 and are presented below the blots. For all tissues, a striking reduction of the expression was detected between E18 and postnatal day 1. This decrease was even more marked in the following post-natal stages. Statistical analyses were performed between E18 and all other stages (**, p < 0.01). *d: days, w: weeks, m: months*.

We then investigated *Sepn1 *expression in isolated quadriceps, diaphragm and non-skeletal-muscle tissues (brain, liver, kidney and heart) from late embryonic stages (E15, E18) to 15 months of age, with an earlier embryonic stage included for the heart (E13) (Figure 2A). By qRT-PCR, *Sepn1 *expression was detected in all tissues, albeit at barely detectable levels in liver (Figure 2A). Overall, variations in the mRNA levels observed for the different tissues were limited. Nevertheless, we observed a significant decrease in expression between E15 and E18 in the brain and in kidney. In parallel, increased levels were detected between E18 and postnatal day 1 in diaphragm, heart and kidney. Lastly, we showed a two fold increase in expression in the brain, between young (1-6 weeks) and adult mice (3-15 months) and a two fold reduction in kidney, between the first week of life and older mice (3 weeks to 15 months) (Figure 2A). Interestingly, expression in quadriceps and diaphragm did not exhibit the highest levels and appeared mostly constant. Similar results were obtained for skeletal muscles isolated from posterior (soleus + gastrocnemius) and anterior (tibialis anterior + extensor digitorum longus) hind limb compartments (data not shown).

We studied the expression of SelN at the protein level by Western blot, using proteins extracted from isolated tissues from E18 to 15 months of age. Several proteins were tested for protein loading control; actin and GAPDH exhibited the least variability between the various developmental stages (Figure 2B and data not shown). Nevertheless, quadriceps homogenates displayed an increase of both actin and GAPDH expressions after 15 days of age; for this tissue, no ideal protein normalization could be obtained. For all the tissues considered except liver, we observed a striking reduction in SelN expression between E18 and postnatal day 1 (about three fold reduction), and even more so at adult ages when it became barely detectable (less than 10% compared to E18) (Figure 2B). In protein extracts from liver, SelN was never detected, even at E18 (Figure 2B), in keeping with the transcript quantifications.

**Figure 3:**
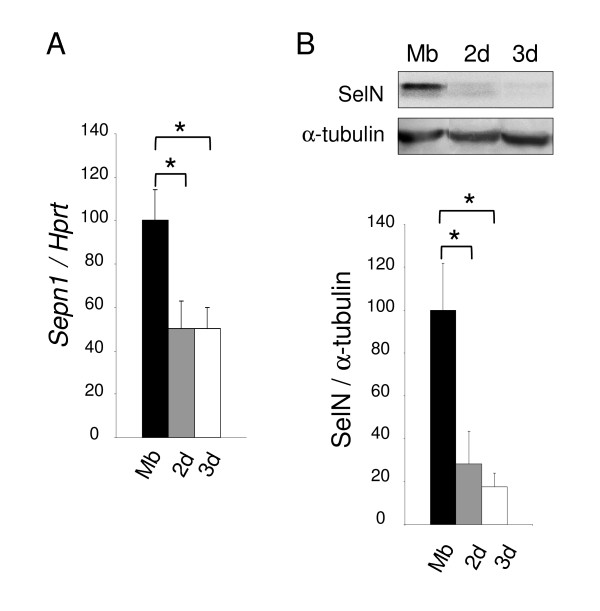
***Sepn1 *expression during murine myoblast differentiation**. **A: ***Sepn1 *qRT-PCR on cDNA from C2C12 myoblasts (Mb) and myotubes after 2 and 3 days (2 d/3 d) in differentiation medium. *Hprt *served for normalization. A two fold reduction of *Sepn1 *expression was observed in myotubes compared to undifferentiated cells. **B: **SelN Western blot analysis on proteins from C2C12 cells (Mb, 2 d, and 3 d), normalized to α-tubulin. Note that the decrease of SelN expression between myoblasts and myotubes is even more marked compared to transcript quantification. *, p < 0.05.

In parallel, using mouse C2C12 muscle cells, we observed a 50% decrease of *Sepn1 *expression in myotubes after 2 and 3 days of differentiation, compared to myoblasts (Figure 3A). This reduction was even more marked at the protein level with a five fold reduction detected in myotubes compared to myoblasts by Western blot (Figure 3B). This is in accordance with data previously obtained with human cells [16].

### Specificity of *Sepn1 *probes

**Figure 4:**
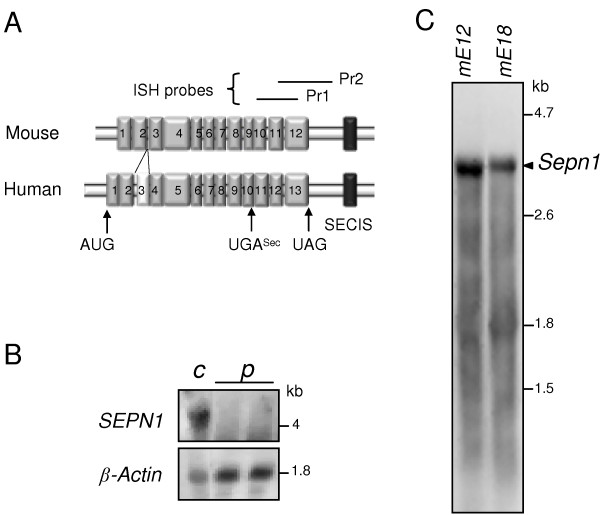
**Specificity of the *Sepn1 *probes**. **A: **Schematic representation of the murine (*Sepn1*) and human (*SEPN1*) mRNA. Exons and the SECIS sequence are represented by grey and black boxes, respectively. The human third exon, corresponding to an Alu sequence, is represented by a white box. Target sequences of Pr1 and Pr2 ISH probes are shown above the schema. **B: **Northern blot analysis, using the Pr1 probe, of RNA extracted from human control fibroblasts (*c*) and fibroblasts from a *RSMD1 *patient with a homozygous premature stop codon, leading to *SEPN1 *mRNA degradation (*p*). *β-actin *is used as a reference. A 4.2/4.3 kb band was observed in control fibroblasts but not in *RSMD1 *fibroblasts, validating the specificity of Pr1 for *SEPN1 *transcripts. **C: **Northern blot analysis of RNA from mouse E12 (*mE12*) and E18 (*mE18*) whole embryos shows a single 3.4 kb band corresponding to *Sepn1 *transcript.

Since no appropriate antibody was available for immunohistochemistry, we investigated *Sepn1 *expression pattern during early embryogenesis by whole mount *in situ *hybridization (ISH). The murine *Sepn1 *cDNA [GenBank: NM_029100], 3461 bp in size, contains 12 exons, the in-frame UGA codon being located in exon 9, and leads to a protein of 557 residues (Figure 4A). In mouse, no sequence homology was found with the alternatively spliced third exon described in human, since this exon corresponds to a primate specific Alu sequence [11]. Two *Sepn1 *probes (Pr1: 573 bp and Pr2: 696 bp) were synthesized from PCR fragments corresponding to the 3' region of the cDNA (Figure 4A). The corresponding murine transcript regions shared more than 85% identity with the human sequence. We performed Northern blot analysis with Pr1 and Pr2 probes, using RNA extracted from cultured human fibroblasts from a control subject and a *RSMD1 *patient with *SEPN1 *mRNA degradation due to a premature termination codon (p.L482fs [20] - Figure 4B). For both probes, one band corresponding to *SEPN1 *transcripts was observed in control RNA, but was undetectable in RNA extracted from the patient, thus validating the specificity of the probes (Figure 4B and data not shown). Using mouse RNA extracted from E12 and E18 whole embryos, we detected only one 3.4 kb band by Northern blot, which corresponds to the predicted transcript size for *Sepn1 *(Figure 4C).

### Expression pattern of *Sepn1 *during embryogenesis

**Figure 5:**
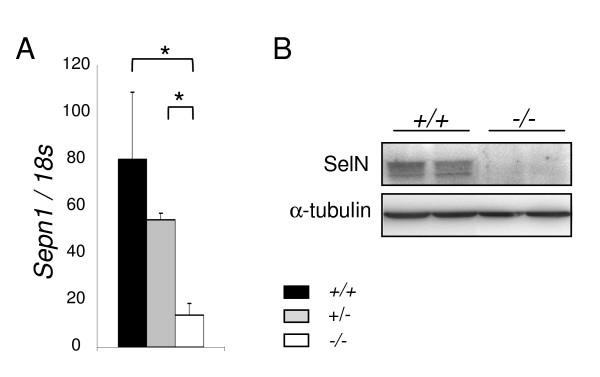
**SelN deficiency in the murine model**. *Sepn1 *expression analyzed by qRT-PCR (A) and Western blot (B) performed on whole E12.5 littermate embryos: wild-type (+/+), heterozygous (+/-) and homozygous mutants (-/-). *18s *gene and α-tubulin were used for normalization. *Sepn1 *transcript expression was almost abolished in the homozygous mutants and SelN was undetectable by Western blot in these mice. *, p < 0.05.

*Sepn1 *deficient mouse embryos were used as negative controls for whole mount ISH. The recombinant gene construct introduced a frame-shift and a premature stop codon within the coding sequence, leading to a loss of the functional protein in mutants (Rederstorff et al., manuscript in preparation). As shown in Figure 5A, *Sepn1 *expression was barely detectable at the transcript levels in E12.5 mutant whole embryos (*Sepn1*^-/-^) compared to wild-type (*Sepn1*^+/+^), indicative of the *Sepn1 *mRNA degradation in the model. At the protein level, SelN was observed in wild-type embryos by Western blot but was undetectable in mutant samples (Figure 5B).

**Figure 6:**
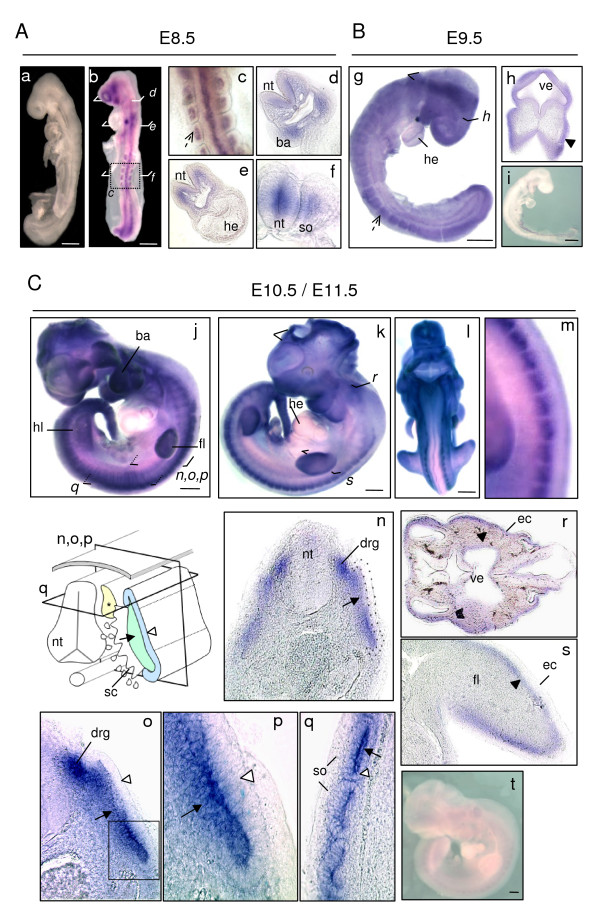
***Sepn1 w*hole mount *in situ *hybridization between E8.5 and E11.5**. **A: **E8.5 wild-type embryos hybridized with sense (a) and antisense (b-f) *Sepn1 *Pr1 probes. No signal was detected with sense probes (a). With the antisense Pr1 probe, all the somites appeared strongly stained (**broken arrow **- panel c). Sectioning with a vibratome revealed expression also in the branchial arches (d) and the neural tube (f). Note that no signal was observed in the heart (e). **B: **E9.5 wild-type (g, h) and *Sepn1*^-/- ^(i) embryos hybridized with the *Sepn1 *antisense Pr1 probe. **Broken arrow **in (g) shows stained somites. Mesenchymal staining (**black arrowhead**) was revealed within vibratome sections (h: section in the forebrain). No signal was seen within the mutant embryos (i). **C: **E10.5 (j) and E11.5 (k-m) wild-type embryos hybridized with the *Sepn1 *antisense Pr1 probe. Dorsal region of wild-type embryos is shown in (m). **(n-s) **High magnification of vibratome sections performed in the trunk (n-q), the head (r), and the limb bud (s). Inset p corresponds to a higher magnification of the outlined region in o. The section plans are indicated in the schema. *Sepn1 *expression was detected in the sub-ectodermal mesenchyme (**black arrowhead**), the myotome (**arrow**) and the dorsal root ganglia (*). Note that overt staining was not observed in the dermomyotome (**white arrowhead**) nor in the heart. No signal was detected in *Sepn1*^-/- ^embryos hybridized with *Sepn1 *antisense probe (t). ba: branchial arches; drg: dorsal root ganglia (*); ec: ectoderm; fl: forelimb; he: heart; hl: hindlimb; nt: neural tube; so: somite; ve: ventricle. Scale bars in (A) = 200 μm, in (B, C) = 400 μm.

Identical expression patterns were obtained with Pr1 and Pr2; results presented in Figure 6 correspond to ISH performed with the Pr1 probe. At the different embryonic stages tested, no signal could be detected using either sense probes with wild-type embryos (Figure 6A, panel a and data not shown) or antisense probes with *Sepn1*^-/- ^embryos (Figure 6B, panel i and 6C, panel t). This confirmed the specificity of the staining pattern obtained.

In E8.5 whole embryos, we detected *Sepn1 *expression in somites and the neural tube, as well as in the mesenchyme located under the ectoderm, in the head and the branchial arches of embryos (Figure 6A, panels b-f). At E9.5, a ubiquitous-like staining was observed in whole embryos, with a stronger expression in somites (Figure 6B, panel g). Upon vibratome sectioning, the staining was associated with expression in the sub-ectodermal mesenchyme, as depicted in forebrain (Figure 6B, panel h). At E10.5 and E11.5, whole embryos appeared strongly stained in the dorsal part of the body, the limb buds, the branchial arches and the head (Figure 6C, panels j-m). Following sectioning, we observed expression in the dorsal root ganglia (drg). Furthermore, within the somites, the myotome appeared strongly stained, whereas little to no signal was detected in the dermomyotome of embryos (Figure 6C, panels n-q). Moreover, as previously seen, the mesenchyme located under the ectoderm exhibited staining, particularly in the head and limb buds (Figure 6C, panels r, s), although this signal was weak and required a longer revelation time as compared to the staining observed in the myotome and drg.

Interestingly, at all of these stages, we did not observe staining in the heart by ISH, whereas *Sepn1 *expression was detected as early as E13 in this tissue by qRT-PCR. This could result either from *Sepn1 *cardiac expression appearing after E11.5, or more likely from the higher sensitivity of the qPCR technique.

### Somitogenesis in *Sepn1 *deficient murine embryos

**Figure 7:**
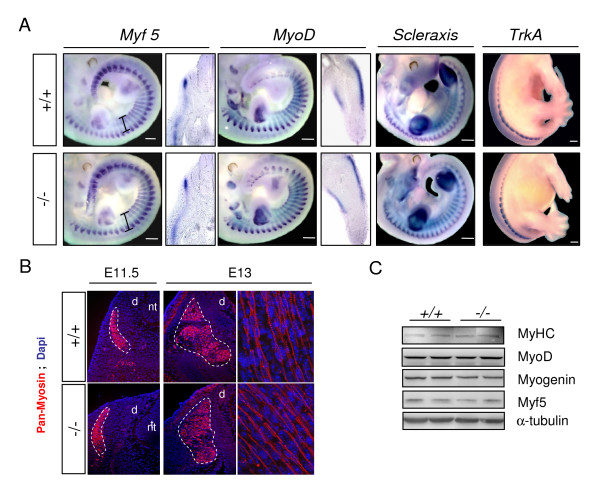
**Somitogenesis in wild-type and *Sepn1*^-/- ^embryos**. **A: **Whole mount *in situ *hybridization, and corresponding vibratome sections, against *Myf5, MyoD, Scleraxis *(E11.5) and *TrkA *(E13) performed on *Sepn1*^+/+ ^and *Sepn1*^-/- ^embryos. No difference was observed in somite organisation and size (black fragments) between wild-type and mutants embryos. The expression pattern of the tested factors appeared unaltered in absence of SelN. Vibratome sections correspond to the trunk level for *Myf5 *and to the limb bud for *MyoD*. **B: **Immunostaining against myosin heavy chain on E11.5 and E13 wild-type and deficient embryos. The expression patterns were identical between the embryos. Myotome is outlined with a white broken line. **C: **Western blot analysis against myogenic factors, performed on proteins from wild-type and deficient E12.5 whole embryos, revealed no alteration of their expression in the mutant embryos. α-tubulin was used for normalization. Scale bars = 400 μm. *d: drg; nt: neural tube*.

Since an early expression of *Sepn1 *was observed in somites and defects were reported in somite organisation and myogenic factors expression in *sepn1 *morpholino injected zebrafish embryos [17,18], we specifically investigated these events in *Sepn1*^-/- ^embryos. At mid-gestation stages (E10 and E11.5), ISH performed against the myogenic determination factors *Myf5*, *MyoD*, and *Pax3 *revealed no defect in somite size and organisation in *Sepn1*^-/- ^embryos compared to wild-type (Figure 7A and data not shown). Moreover, the expression pattern of these factors was similar in both types of embryos (Figure 7A). Likewise, the expression of myosin heavy chain, a marker of differentiated muscle cells, was unaltered when visualized by immunohistochemistry in E11.5 and E13 deficient embryos (Figure 7B). These data were confirmed by qRT-PCR and Western blot analyses that demonstrated no modification of the myogenic factors expression in the deficient embryos (Figure 7C and data not shown). Lastly, the expression patterns of *Scleraxis*, involved in the establishment of myotendinous junctions [21], and of *TrkA*, a neurotrophin receptor which was used as a marker of the drg [22], appeared unmodified in *Sepn1*^-/- ^embryos by whole-mount ISH (Figure 7A).

## Discussion

In this study, we characterized for the first time the precise spatio-temporal expression pattern of the murine *Sepn1 *gene, the homolog of *SEPN1 *involved in several forms of early onset myopathies in humans. We showed that *Sepn1 *is expressed early during embryogenesis and strongly down-regulated before birth. We also demonstrated that, in most isolated tissues, *Sepn1 *expression exhibited limited variations at the transcript levels between pre- and post-natal stages, but significant changes at the protein levels were detected, thereby pointing to a major post-transcriptional regulation of this gene. Of particular interest are some specific tissues where a significant increase in the expression of transcript was observed between E18 and postnatal day 1 (for kidney) or older mice (for the brain), whereas an almost 80% decrease in the protein levels was detected between equivalent stages. Despite the lack of data concerning the developmental expression pattern of Sec insertion factors, one regulatory mechanism, inherent to selenoproteins, may be based on the availability of these specific factors. Indeed, this complex machinery may exhibit spatial and temporal regulation, associated with a hierarchy in the synthesis of selenoproteins, leading to potential fine tuning of each selenoprotein in both physiological and selenium deprivation conditions [23-26]. It is thus conceivable that SelN expression is modulated depending on its requirements and biological roles in each tissue at the various developmental stages. Notably, we reported recently that the loss of interaction between the SECIS sequence and SBP2 abolished SelN translation and also led to the degradation of *SEPN1 *mRNA [20]. Therefore, modifying the interaction with the translational machinery may also affect *Sepn1 *transcript stability in mouse. Hence, whether the regulation of SelN during development depends on the availability of Sec insertion factors remains questionable.

Another regulatory pathway could involve microRNAs, recently reported as major factors for translation modulation without mRNA expression perturbation [27]. Their importance in several developmental mechanisms has already been well established in most species, including vertebrates [28]. We determined that the *Sepn1 *sequence is a potential target for several microRNAs (PC, PG, personal communication); however, their biological relevance and developmental involvement are not yet established. On the other hand, SelN expression may also be modulated by protein degradation or cleavage, as reported for different proteins located within the endoplasmic reticulum membrane [29]. Such proteolysis was suggested in the study of Deniziak et al. who observed a potential cleavage product by Western blot that correlates with reduced expression of SelN in zebrafish [18]. However, it was not investigated whether the degradation was physiological, or rather linked to the experimental conditions. Lastly, it is noteworthy that in some tissues, such as kidney and liver, in which similar dynamics were observed for protein and transcript expression, the gene is most likely regulated at the transcriptional level, although the factors involved are not known yet. Taken together, our findings reveal that complex transcriptional and translational regulation networks may be associated with the expression of SelN during development.

The expression pattern presented herein provides interesting insights into the physiological functions of SelN. Indeed, in all of the tissues examined except liver, high levels of SelN expression were limited to a particular temporal window, with a pronounced and progressive reduction observed between E18 and post-natal stages. These results are consistent with those obtained in zebrafish where a strong reduction of the expression was detected during somites differentiation [17,18], and in humans where a decrease was observed between foetal and adult tissues [16]. This developmental pattern suggests that SelN may participate in the maturation of cells and organs from mid-gestation to perinatal stages. This notion is supported by the observation that SelN expression decreased during both human [16] and murine myoblast differentiation in culture. To our knowledge, there is no similar expression pattern reported for other selenoproteins but this may be at least partly due to the lack of studies focusing on the expression pattern of these proteins in mammals. Notwithstanding, in mouse, selenoprotein W (SelW) was found to be expressed early during development (E6) and to decrease during myoblast differentiation, but comparisons between pre- and post-natal stages in different tissues were not reported [7]. Moreover, in zebrafish, SelM was the only other selenoprotein reported to be expressed at the transcript levels in somites [6].

Based on the previous data obtained in humans and zebrafish, a potential link between SelN and cell proliferation has already been proposed [16]. At mid-gestation stages, we found that *Sepn1 *is expressed in the mesenchyme located under the ectoderm. Taking into account our previous work showing high expression in fibroblasts [16], we attributed this expression to mesenchymal cells that share the general features of fibroblasts. Moreover, we first hypothesized that this pattern could correlate with a higher proportion of proliferative cells in this area. However, BrdU staining in E11.5 embryos did not reveal a higher mitotic activity in this region (data not shown). Likewise, in mature somites, *Sepn1 *expression was detected in the myotome, mainly composed of post-mitotic cells that have initiated the myogenic program, while its expression was almost undetectable in the dermomyotome where most cells are proliferating [30]. Therefore, at least during mouse embryogenesis, there is no straightforward correlation between *Sepn1 *and cell proliferation.

The early expression detected in somites correlates with ISH results obtained in zebrafish [6]. Moreover, in mice, the restricted expression found in myotome indicates that *Sepn1 *is expressed by muscle precursors and maturating myocytes but less or not by uncommitted cells. Interestingly, in contrast to observations performed on zebrafish embryos knock-down for *sepn1 *expression [17,18], we did not detect any defects in somite organisation nor in myogenic factors expression, at mid-gestation stages, in *Sepn1 *mutant embryos. This suggests that SelN is dispensable for somitogenesis as well as the early waves of myogenesis in mouse. The simplest explanation would be that redundant activities exist in mouse, which may be less active in others animals. Due to the common reactivity of selenoproteins conferred by the Sec residue, one can hypothesize that some functional redundancy may exist within the protein family. Therefore, it would be interesting to further investigate other selenoproteins located in the endoplasmic reticulum, such as Sep15, SelS and SelM, and/or expressed predominantly during embryogenesis, like Gpx6 [2]. In addition, among the selenoproteins expressed in skeletal muscles, SelW is particularly interesting because of its dynamic expression during muscle maturation and its presumed association with white muscle disease [31]. Quantification of several selenoproteins expression by qRT-PCR revealed no difference between E12.5 wild-type and mutant *Sepn1*^-/- ^whole embryos (data not shown). However, additional experiments, including older mice and protein analyses, need to be performed to resolve this issue.

Lastly, the barely detectable levels of SelN expression found in mature muscles fit with the previous observations in human tissues [16] and suggest a limited role for the protein at these stages. This contrasts with the progressive evolution of the pathology with age. One reason may be that SelN deficiency during muscle development leads to increased fragility of the tissue that worsens during aging. Another non necessarily exclusive hypothesis is that SelN is required in mature muscles subjected to constant tonic contractions, since the pathology affects more severely the respiratory and postural muscles. However, our data showing similar expression levels in muscles as different as quadriceps and diaphragm do not appear to sustain this hypothesis. The characterization of *Sepn1 *deficient mice, from later developmental stages to adult ages, will constitute a major point of investigation in order to better understand SelN function in muscle physiology and the remaining paradox regarding its almost ubiquitous expression and the specific muscular phenotypes.

Taking into account the recent studies suggesting a role for SelN in the regulation of ryanodine receptors (RyR) activity [17,19], it is interesting to try and correlate their developmental activity with the expression pattern of SelN described herein. RyR1 expression was reported in mouse myotome as early as E9.5 by ISH [32] but significant protein expression was only detected at late embryonic stages in rodents [33,34]. In addition, RyR1 expression greatly increases two weeks after birth and becomes predominant in mature muscles, therefore differing from SelN pattern [33-35]. In contrast, RyR3 expression strongly decreases at postnatal day 15 and it is almost undetectable in most adult tissues [33-36]. Furthermore, it was reported that its expression is exclusively maintained in highly active muscles, such as diaphragm or neck muscles [37] in which it may amplify calcium signals [38,39]. Interestingly, these tissues correspond to the most affected muscles in *SEPN1*-related myopathies. Taken together, this comparison between RyR channels and SelN weakens the proposed functional link between RyR1 and SelN, but suggests a closer relationship between RyR3 and SelN. This is further substantiated by our ISH results. Indeed, *Sepn1 *staining was detected in the dorsal root ganglia which correspond to neural crest derivatives containing cell bodies of sensitive neurons, where a CICR mechanism was reported and associated with RyR3 channels [40,41]. We note also that *Sepn1 *was expressed in the sub-ectodermal mesenchyme, a region known to dynamically participate in cell migration during development, with active calcium waves involved in this process. Finally, the precise role of SelN in the regulation of RyR channels and the existence of other substrates remain largely unsolved.

## Conclusion

This is the first study that establishes the precise spatio-temporal expression pattern of selenoprotein N in mammals, with both transcript and protein analyses at the different developmental stages. We showed that SelN is expressed early during mouse embryogenesis, particularly in somites and muscle precursors. Nevertheless, it appears to be dispensable for correct somitogenesis and expression of myogenic factors at mid-gestation stages. We also demonstrated that SelN expression pattern is wide-spread and is subject to post-transcriptional regulations which lead to the significant reduction of its protein expression levels during the perinatal period. Our results suggest important functions for SelN during the maturation of cells and organs. Further investigations should focus on the particular developmental window when SelN is strongly expressed, to determine the involvement of this selenoprotein in muscle physiology and to unveil the pathophysiological mechanisms leading to muscle alteration in *SEPN1*-related myopathies.

## Methods

### Cell culture

Mouse C2C12 cells were grown in an humidified atmosphere of 5% CO_2_, at 37°C, in Dulbecco's modified Eagle's medium (DMEM, Invitrogen) supplemented with 20% foetal calf serum (FCS, Invitrogen), 2 mM gentamycine (Invitrogen). Differentiation into myotubes was induced by switching the medium to DMEM supplemented with 1% FCS.

### Mice and genotyping

C57Bl/10 mice were euthanized with isoflurane and tissues were dissected at various ages. Embryos were obtained from gestating females of the *Sepn1 *knock-out lineage (Rederstorff et al., article in preparation) between embryonic days 5.5 (E5.5) and 18.5 (E18.5), the morning of the vaginal plug indicating the stage E0.5. For *in situ *hybridization, embryos were dissected at 4°C in phosphate buffer saline (PBS) and freed from their extra-embryonic membranes. At E9.5 and older stages, the neural tube was pierced open at the level of the brain to facilitate exchange of solutions. Embryos were fixed overnight at 4°C, in 4% paraformaldehyde (PFA), dehydrated in methanol and conserved at -20°C. DNA extraction was carried out for genotyping with the Wizard Genomic DNA Isolation System (Promega) according to the manufacturer's recommendations. All animal studies were performed in accordance with the European Union guidelines for animal care and approved by the local ethical committee on animal experimentation.

### Total RNA extraction and first-strand cDNA synthesis

Whole embryos and isolated tissues were frozen in liquid nitrogen. Total RNA was extracted using the RNeasy Fibrous Tissue Mini Kit (Qiagen) according to the manufacturer's instructions. Total RNA was also extracted from cultured human fibroblasts (control and *RSMD1 *patient with a c.1446delC homozygous mutation) by Trizol (Invitrogen) procedure as previously described [11]. RNA yield and quality were assessed with the Nanodrop system (Thermo Scientific) and on the 2100 Bioanalyzer (Agilent Technologies). RNA samples with a RIN (RNA Integrity Number) superior to 8 were used for quantification. cDNA was synthesized from 500 ng of RNA with random hexamer primers using the SuperScript First-Strand Synthesis System for RT-PCR (Invitrogen).

### Quantitative PCR (qPCR)

*Sepn1 *transcripts were quantified by real time PCR using the following primers: 5'GCTTTCCTGTAGAGATGATG3'/5'GCCCCGCCGGAGTCCTTC3'. qPCR were performed on the LightCycler480 System (Roche) using the LighCycler480 SYBR Green I Master mix (Roche). The program included an initial denaturation step of 8 min at 95°C, followed by 40 amplification cycles of denaturation at 95°C for 10 sec, hybridization at 63°C for 15 sec, and elongation at 72°C for 15 sec. Quantification of additional genes was performed similarly with the following primers: 18s: 5'ACCTGGTTGATCCTGCCAGT3'/5'CTCACCGGGTTGGTTTTGAT3'; *Hprt: *5'AAGCAGATGGCCACAGAACT3'/5'ACCCCACGAAGTGTTGGATA3'. Data were analyzed using the LightCycler480 analysis software (Roche).

### RNA probes synthesis and whole-mount In Situ Hybridization (ISH)

Two digoxigenin (DIG) labeled riboprobes (Pr1/Pr2) were generated from *Sepn1 *PCR fragments obtained from total murine cDNA with the following primers: Pr1: 5'TCCTAGATGAGGACGGCAAC3'/5'GGTCCTCAAAGCTGGATGAG3'; Pr2: 5'CGCCCATCCTCACTCTCCTC3'/5'TCCAGTCCACTCCCTATTCACA3'. Fragments were cloned in the pGEM-T vector (Promega). Antisense and sense probes were synthesized using the DIG RNA Labeling Kit (Roche) with T7 or SP6 RNA polymerase. Sense probes were used as negative controls. Yield and quality of the probes were estimated by electrophoresis and dot blot compared to a standard (β-actin probe - Roche). *MyoD*, *Myf5, Scleraxis *and *TrkA *probes, previously described [42,43], were also used for whole mount ISH.

### Whole embryo In Situ Hybridization

*In situ *hybridizations (ISH) were performed on embryos aged from E8.5 to E11.5, as previously described [43]. Briefly, after rehydration, embryos were treated with 10 μg/ml proteinase K (Qiagen) at room temperature, from 10 to 30 min according to their age and fixed in 4% PFA. Embryos were incubated for more than 1 h in hybridization buffer (50% formamide, 1.3× SSC pH 5, 50 μg/ml yeast RNA, 5 mM EDTA pH 8, 0.2% Tween-20, 0.5% CHAPS, 100 μg/ml heparin) and then overnight with the appropriate probe at 0.5 to 2 μg/ml, at 69°C. Embryos were successively washed in hybridization buffer, in 1:1 hybridization buffer/MABT (100 mM maleic acid, 150 mM NaCl, 1% Tween-20), and finally in MABT. After blocking in MABT, 2% Blocking Reagent (Roche), 20% inactivated goat serum (Invitrogen) for more than 1 h at room temperature, embryos were incubated overnight at 4°C in alkaline phosphatase conjugated anti-DIG antibody (1:2000 - Roche). Revelation was obtained with the BM Purple solution (Roche) at room temperature and/or at 4°C, during different time laps depending on the probe. Vibratome sections (50 to 80 μm) were performed after inclusion of the embryos in 10% sucrose overnight at 4°C and then in 20% gelatin.

### Northern Blotting

30 μg of total RNA were resolved on 1% formaldehyde agarose gel and blotted overnight onto a positively charged nylon membrane (Roche). RNA were UV cross-linked to the membrane during 3 min. Membranes were hybridized overnight at 68°C with diluted probe (100 ng/ml) in hybridization buffer (DIG Easy Hyb buffer - Roche) and then rinsed in 2× SSC, 0.1% SDS at room temperature and in 0.1× SSC, 0.1% SDS at 68°C. Membranes were blocked in Blocking Reagent (Roche) diluted in 0.1 M maleic acid, 0.15 M NaCl, pH 7.5, and then incubated with alkaline phosphatase conjugated anti-DIG antibody (Roche) in maleic acid buffer. After incubation in detection buffer (0.1 M Tris-HCl, 0.1 M NaCl, pH 9.5), signals were obtained with the CDP-star, ready-to-use buffer (Roche) on the ChemImager system (Alpha Innotech).

### Protein extraction and Western blot analysis

Frozen tissues were ground in liquid nitrogen and homogenized in protein extraction buffer (80 mM Tris-HCl, pH 6.8, 10% SDS, 120 mM sucrose, 10 mM EDTA, 1 mM PMSF and 1 mM benzamidine). Homogenates were incubated 10 min at 55°C, sonicated twice and quantified with the BCA Protein Assay (Pierce). 60 μg of proteins were electrophoretically separated in 8% polyacrylamide SDS gel and transferred to a polyvinylidene difluoride membrane (Invitrogen). Membranes were immunoprobed with the following primary antibodies: rabbit anti-SelN (ab137 [16]), mouse anti-tubulin α (Sigma T5168), rabbit anti-actin (Sigma A2066), rabbit anti-MyoD (Santa Cruz sc-304), rabbit anti-myogenin (Santa Cruz sc-576), rabbit anti-Myf5 (Santa Cruz sc-302) and mouse anti-MyHC (ATCC, MF20). They were then incubated with HRP-conjugated secondary antibodies (Dako). Signals were detected with chemiluminescent HRP substrate (Immobilon Western - Millipore) on the SynGene instrument (Ozyme) and quantified with the ImageJ software.

### Immunohistochemistry

After rehydration, E12.5 whole embryos were equilibrated in 15% sucrose overnight at 4°C. They were then frozen in liquid nitrogen-cooled isopentane. Ten μm cryostat sections were fixed in 4% PFA, rinsed in 0.1 M glycine, and incubated in methanol at -20°C. They were then placed in boiling 0.01 M citric acid for 10 min to achieve antigen retrieval. Sections were blocked in 3% IgG free bovine serum albumin (BSA - Jackson ImmunoResearch) overnight at 4°C and with ChromoPure Mouse IgG, Fab Fragments (Jackson ImmunoResearch) for 30 min. Sections were then incubated in 3% IgG free BSA, 0,1% Triton X-100, with primary antibodies (MyHC: monoclonal mouse ATCC MF20-1/100; laminin: polyclonal rabbit Abcam ab11575-1/1000) for 3 h at room temperature. Alexa Fluor secondary antibodies (goat anti-mouse A568, goat anti-rabbit A488; Jackson ImmunoResearch) were incubated on slides for 90 min at room temperature. Sections were rinsed, mounted in Vectashield DAPI (Vector) and observed with an Axiophot microscope (Zeiss). Images were captured using the Metaview software (Ropper Scientific).

### Statistical analyses

Results represent Means ± SD. Comparisons between two groups were performed using a Student's *t*-test or a Mann-Whitney rank sum test depending on the normal distribution of data, with a 0.05 level of confidence accepted for statistical significance. Multiple group comparison was performed by one way ANOVA with Dunn's pairwise procedures (p < 0.05).

## Abbreviations

*CFTD*: Congenital Fibre Type Disproportion; CICR: Calcium-Induced Calcium Released; drg: dorsal root ganglia; eEFSec: eukaryote Elongation Factor; ISH: *In situ *Hybridization; *MB-DRM*: Desmin-Related Myopathy with Mallory Body-like inclusions; *MmD*: Multi-minicore Disease; qRT-PCR: quantitative Reverse Transcription - Polymerase Chain Reaction; *RSMD1*: Rigid Spine Muscular Dystrophy; RyR: Ryanodine Receptor; SBP2: SECIS Binding Protein 2; SD: Standard Deviation; Sec: Selenocystein; SECIS: Sec Insertion Sequence; SelN: Selenoprotein N; *SEPN1*-RM: *SEPN1 *Related-Myopathies.

## Authors' contributions

PC carried out the qPCR, western blotting and *in situ *hybridization, analyzed and interpreted the data and drafted the manuscript. SM performed ISH probes synthesis. CG participated to the qPCR studies, and purified SelN antibodies. MR, AL and AK constructed and provided the deficient murine model. ST helped on ISH procedures and analysis of the results. VA and PG conceived and coordinated the overall study and helped draft the manuscript. All authors read and approved the final manuscript.
